# A Modified TALEN-Based Strategy for Rapidly and Efficiently Generating Knockout Mice for Kidney Development Studies

**DOI:** 10.1371/journal.pone.0084893

**Published:** 2014-01-08

**Authors:** Yunhong Liu, Xiaoyan Lv, Ruizhi Tan, Tianming Liu, Tielin Chen, Mi Li, Yuhang Liu, Fang Nie, Xiaoyan Wang, Puhui Zhou, Mianzhi Chen, Qin Zhou

**Affiliations:** 1 Core Facility of Genetically Engineered Mice, Regenerative Medicine Research Center, West China Hospital, West China Medical School, Sichuan University, Chengdu, Sichuan, China; 2 Department of Dermatology, West China Hospital, West China Medical School, Sichuan University, Chengdu, Sichuan, China; 3 Lab of Molecular Nephrology, Chongqing Medical University, Chongqing, China; 4 State Key Laboratory of Biotherapy and Cancer Center, Sichuan University, Chengdu, Sichuan, China; National Cancer Institute, United States of America

## Abstract

The transcription activator-like effector nucleases (TALENs) strategy has been widely used to delete and mutate genes in vitro. This strategy has begun to be used for in vivo systemic gene manipulation, but not in an organ-specific manner. In this study, we developed a modified, highly efficient TALEN strategy using a dual-fluorescence reporter. We used this modified strategy and, within 5 weeks, we successfully generated kidney proximal tubule-specific gene *Ttc36* homozygous knockout mice. Unilateral nephrectomy was performed on the 6-week-old founders (F0) to identify the knockout genotype prior to the birth of the offspring. This strategy was found to have little effect on reproduction in the knockout mice and inheritability of the knockout genotypes. The modified TALEN knockout strategy in combination with unilateral nephrectomy can be readily used for studies of gene function in kidney development and diseases.

## Introduction

Genetic manipulations in genetically engineered mice have been widely used to study gene function and understand the roles of genes in human genetic diseases. Currently, there are a variety of genetic or genomic manipulation technologies available, including the custom-made transcription activator-like effector nucleases (TALENs). TALENs are precise and efficient genomic engineering tools, which have been successfully used in many fields of scientific research [1]. They are fusion proteins and work in pairs, consisting of a modular DNA-binding domain and a FokI endonuclease monomer [2], [3]. When two TALENs bind to their DNA targets, the FokI monomers will dimerize and introduce a DNA double-strand break within the specific binding site [4]. The DNA break can either be repaired by non-homologous end-joining (NHEJ) or homologous recombination (HR), which results in deletion/insertion mutations and specific site mutations or specific sequence additions, respectively. This strategy has the potential to directly edit the genome of one-cell mouse embryos, which is a superior strategy compared to the traditional and time-consuming gene knockout strategies that are involved in gene-targeting vectors, embryonic stem (ES) cells, and chimaera selection [5], [6]. However, the conventional TALENs screening systems are mainly based on β-galactosidase or a single fluorescence reporter [7], [8], which functions in an indirect manner and limits the assessment of the transfection efficiency.

TALENs have recently begun to be used for gene manipulation in vivo, but not in the field of kidney development and diseases [5], [9]–[11]. Here, we modified the TALENs gene manipulation strategy using a dual-fluorescence reporter, and generated the renal proximal tubule gene, *Ttc36* [12], with an average mutagenesis efficiency of 51.3% in knockout mice. We also demonstrated that unilateral nephrectomy can be used for rapid identification of knockout mice prior to birth. Moreover, we showed that unilateral nephrectomy has little effect on reproduction in knockout mice and inheritability of the knockout genotypes. In addition, we have shown that cytoplasm microinjection gave rise to a mutagenesis efficiency of 85.7% compared with 30.4% in the pronucleus, which makes better understanding of TALENs-induced homologous recombination in the nucleus.

## Materials and Methods

### Ethics Statement

All mouse surgeries were performed under sodium pentobarbital anesthesia. Animals were treated according to the recommendations in the Guide for the Care and Use of Laboratory Animals of the National Institutes of Health. The experimental procedures were approved by the Animal Experimental Ethics Committee of Sichuan University.

### TALENs Construction

**Figure 1:**
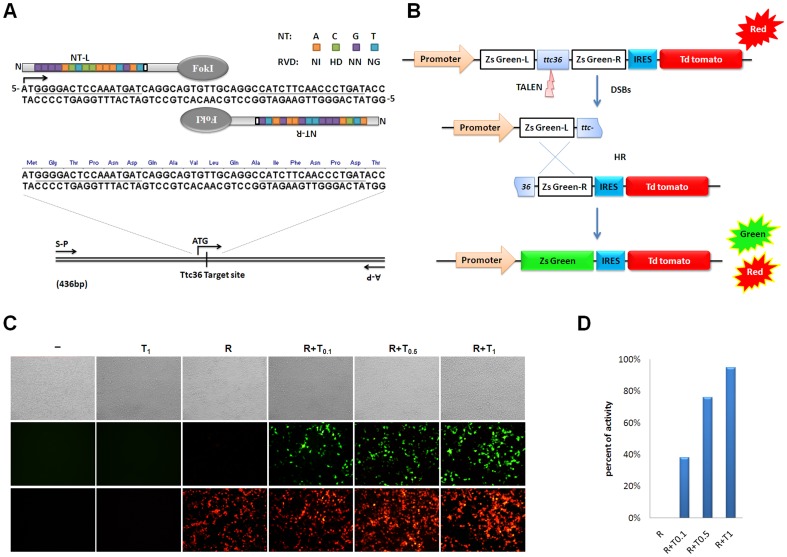
TALENs structure and nuclease activity. (**A**) Schematic of TALENs protein and the mouse targeted mouse targeted *Ttc36* loci. Left (NT-L) and right (NT-R) TALENs DNA-binding sequences (underlined, 16 bp) with the spacer region (17 bp) in the TALEN-*Ttc36* pair were fused to the FokI endonuclease catalytic domain. The targeted site and primers (S-P, A-P) amino acid sequences for *Ttc36* genomic PCR are also shown. (**B**) Outline of the nuclease reporter assay. TALEN-*Ttc36* targeted sequences were cloned into the locations between the report plasmid ZsGreen gene segments. Reporter plasmid co-transfection of the TALEN-*Ttc36* vectors into HEK293FT cells led to nuclease dependent gene repair and ZsGreen was expressed, as shown by green fluorescence. The red fluorescence from tdTomato was used as an indicator of plasmid transfection efficiency. (**C**) Fluorescence images of the HEK293FT cells at 48 h after transfection. Each well was photographed 3 times: in white, green and red. “-”, without plasmid transfection; R, 0.5 ug/ml reporter plasmid; T0.5, 0.5 ug/ml TALEN-*Ttc36* vectors; T0.1, 0.1 ug/ml TALEN-*Ttc36* vectors; T1, 1 ug/ml TALEN-*Ttc36* vectors. Negative control: 0.5 ug/ml reporter plasmid only. Image magnification: ×200. (**D**) TALEN-*Ttc36* reporter activity. T1+R showed the highest activity (∼ 95%) compared to activities of reporter plasmids with different TALEN-*Ttc36* vector concentrations.

To design an appropriate TALEN targeting site and to allow convenient identification, a targeted sequence near the ‘ATG’ code within the first exon of *Ttc36* (accession number: NM_138951.1) was chosen. TALEN-Left (NT-L) was designed against the sequence (5′ GGGGACTCCAAATGAT3) for the sense strand, and TALEN-right (NT-R) TALENs was designed against the sequence (5′ ATCAGGGTTGAAGATG) for the antisense strand. The pair included bipartite 16-bp targeted sequences separated by a spacer region of 17 bp (caggcagtgttgcaggc; Figure 1A). Plasmids encoding TALENs were constructed using the Golden Gate TALEN Assembly kit (Addgene, cat #1000000016) and according to the code rules as follows: HD for C, NG for T, NI for A, and NN for G [13]. The TALENs repeat-variable diresidue domain (RVD) arrays were then sub-cloned into pCS2+ expression vectors, which harbor the FokI nuclease (Sequence S1 in Supporting Information). The TALEN*–Ttc36* plasmids were confirmed using DNA sequencing analysis.

### Reporter Plasmid Construction

Using PCR, the right segment of the ZsGreen gene was amplified from pLVX-IRES-ZsGreen Vector (Clonetech Laboratories) using the primers TLN-rep-F1 (5′-CCGGAATTCATGGCCCAGTCCAAGCACGGCCTG-3′) and TLN-rep-B1 (5′-CCGCTCGAGTCAGTTGTCGGTCATCTTCTTCATCAC-3′), and cloned into pLVX-IRES-tdTomato Vector (Clonetech Laboratories) at EcoRI - XhoI sites. The left segment of the ZsGreen gene was amplified using PCR from pLVX-IRES-ZsGreen Vector using the primers TLN-rep-F2 (5′-GCTCTAGATGACCATGAAGTACCGCATGGAG-3′) and TLN-rep-B2 (5′-CGCGGATCCTCAGGGCAAGGCGGAGCCGGAGGCGATG-3′), and cloned into pLVX-IRES-tdTomato Vector at XbaI - BamHI sites. The TALEN gene sequence targeting the gene *Ttc36* region (558 bp) was inserted into restriction sites at XhoI - XbaI.

### Fluorescence analysis of the TALEN Activity

HEK293FT cells were cultured in Dulbecco's Modified Eagle Medium (DMEM High Glucose, Gibco) supplied with 10% FBS (vol/vol, Gibco). TALEN reporter plasmid (5 µg) and 1 µg, 5 µg, or 10 µg of each TALEN expression plasmid were co-transfected into a total of 1 × 10^6^ cells. A transfection control was performed using 5 µg TALEN reporter and 10 µg TALEN expression plasmid. Fluorescent images were obtained using a fluorescence microscope (Olympus IX71) at 24 h and 48 h after transfection. The images were taken at 200X magnification, and the fluorescence spots were counted using Image J software (1.44p, NIH). The numbers of cells with red fluorescence were divided by the numbers of cells with green fluorescence, indicating the TALENs activity.

### One-Cell Embryos Microinjection and Genotyping

TALEN mRNA was prepared using the MEGAscript® SP6 Kit (Ambion, Life Technologies) in vitro and purified using the RNeasy Mini Kit (Qiagen), according to manufacturer's instructions. The TALEN*-Ttc36* mRNA was diluted to a working concentration of 10 ng/µl in injection buffer (10 mM Tris, 0.1 mM EDTA, pH 7.4). The mixture should be blended and stored at −80°C. For gene targeting, zygotes were obtained from C57BL/6 mating females and injected with a mixture of the TALEN mRNAs. Microinjections were performed using a micromanipulator system (Nikon, NT-88-V3). Injected zygotes were cultured overnight in KSOM medium, and then transferred surgically to pseudopregnant BALB/c foster mothers at 0.5 dpc. The genomic DNA was extracted from mouse tail or kidney using a TIANamp Genomic DNA Kit (Tiangen BioTech, Beijing), and a 436 bp PCR product was acquired using the PCR sense primer (S-P): 5-AACCACCCTGCCAGGTCAGTAACC and antisense primer (A-P): 5-TCAGAGGACCTCTCAGACTCCAGTC (Figure 1A). The PCR products from mouse tails were recovered, cloned and sequenced, while others from kidney were recovered and directly sequenced. All new DNA sequencing data has been deposited in GenBank (Accession number: KF769035 to KF769075).

### Unilateral nephrectomy

Six-week-old mice with a body weight of 20 to 24 g were anesthetized using sodium pentobarbital (50 mg/kg, intraperitoneally). A small lumbar incision was made and the left kidney was exposed and removed. The kidneys from nephrectomized mice were divided into the two parts: one was immediately frozen in liquid nitrogen for mRNA and protein extraction, and the rest was fixed in 4% paraformalin for frozen section and immunofluorescence analysis.

### Semi-quantitative RT-PCR

Total RNA was isolated from part of the left nephrectomized kidney using TRIzol reagent (Life Technologies), according to the manufacturer's instructions, and quantified using a NanoDrop™ 2000 spectrophotometer. Reverse transcription was performed with RevertAid First Strand cDNA Synthesis Kit (Thermo Scientific) and OligoTs, according to manufacturer's instructions. PCR was performed using a TransTaq™ DNA Polymerase High Fidelity (HiFi) kit (Transgene, Beijing). For semi-quantitative RT-PCR, the *Ttc36* primers were 5-GGGACTCCAAATGATCAGGCAGTG (sense) and 5-GCGCCCGTTGCTGGGCGCGCGCAGC (antisense) and Gapdh (from the RevertAid First Strand cDNA Synthesis Kit) was used as an internal reference. The PCR reaction was carried out at 94°C for 4 min followed by 30 cycles of 94°C for 30 s, 58°C for 30 s, and 72°C for 50 s. The PCR products were analyzed in a 1.5% TAE Gel.

### Western blotting

Protein was extracted from part of the left nephrectomized kidney and analyzed in a 4% to 12% SDS-PAGE gel under reduced conditions. After electrophoresis, proteins were transferred to PVDF membrane (0.45 µm, Millipore) and developed with a rabbit anti-mouse TTC36 primary antibody (pAb; diluted 1∶1000, produced in our lab) or an anti-β Tubulin monoclonal antibody (mAb; Santa Cruz Biotechnology), followed by a horseradish peroxidase-conjugated secondary antibody, and visualized using a DAB Substrate Kit (Thermo Scientific).

### Immunofluorescence Staining

Part of the left nephrectomized kidney was fixed in 4% paraformaldehyde, embedded in Tissue-Tek O.C.T Compound (Sakura Finetek USA) and stored at −80°C. The kidneys were cut into 18 µm sections using a cryostat (Leica, CM1850) followed by air drying for 2 h at room temperature. Immunofluorescence staining was performed according to standard procedures. Briefly, sections were incubated with rabbit anti-mouse TTC36 pAb (diluted 1∶500, produced in our lab) overnight at 4°C in a humidified chamber, with Alexa Fluor® goat anti-rabbit 594 as a secondary antibody (diluted 1∶1000, Life Technologies). For proximal tubules identification, FITC-conjugated LTL (dilution 1∶300, Vector Laboratories) were added during secondary antibody incubation. Nuclei were visualized by DAPI (4′,6-diamidino-2-phenylindole, diluted in 1∶5000, Life Technologies). Staining images were taken using a fluorescence microscope (Olympus IX71) and processed using the Image J software (1.44p, NIH).

## Results

### Determination of the TALEN activity using a dual-fluorescence reporter in vitro

To determine the activity of TALEN*–Ttc36* before microinjecting the mouse embryo, we constructed a reporter plasmid based on two fluorescent proteins, ZsGreen and tdTomato (Figure 1B). The TALEN targeted gene region was inserted into a partly duplicated and nonfunctional ZsGreen gene followed by a sequence of internal ribozyme entry sites (IRESs) and a tdTomato gene. TALEN-induced double-strand breaks (DSBs) stimulated the ZsGreen gene segment repair, which converted ZsGreen into a functional reporter gene. Green fluorescence indicated DSBs and HR efficiency, while the tdTomato protein red fluorescence indicated transfection efficiency (Figure 1C). The reporter and different concentrations of TALEN*–Ttc36* expression plasmids were co-transfected into HEK293FT cells. Both green and red fluorescence was measured. The TALEN activity was the result of dividing the number of the red fluorescence cells by the number of green fluorescence cells. We found that TALEN*–Ttc36* plasmids at 1 µg/ml presented the highest nuclease activity (approximately 95%) without any observed cytotoxicity at 48 h after transfection (Figure 1D). This indicated that TALEN*–Ttc36* worked well at the cellar level and was safe for HEK293T cells.

### Generation of homozygous *Ttc36* knockout mice with TALENs

**Figure 2:**
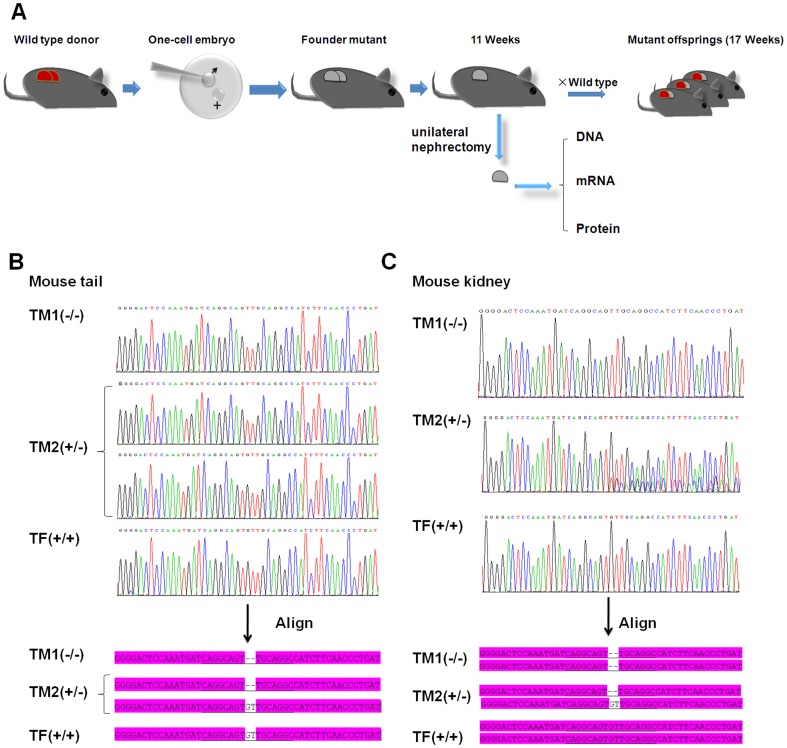
Flow diagram for generation of *Ttc36* mutant mice and DNA sequencing of cleavage sites. (**A**) Schematic of TALEN-mediated kidney-specific gene targeting in one-cell embryos. TALEN-*Ttc36* mRNA microinjections were performed on day 1, knockout mice were generated after 3 weeks, left kidney nephrectomy was performed at 6 weeks, the 3 weeks were allowed for mice to recover from the surgery, and then there were 20 days for development of progeny (4 weeks old). Heterozygous offspring of *Ttc36* mutant founders were obtained within 17 weeks and preliminary study data on the knockout mouse kidney was obtained within 11 weeks. (**B**) DNA sequencing chromatogram of TM1, TM2 and TF tails form genomic DNA PCR products. (**C**) DNA sequencing chromatogram of TM1, TM2 and TF kidneys from directed PCR products. The knockout of “GT” base shown in the alignment was the same in tail and kidney sequencing. TM1, TALEN-*Ttc36* male mouse#1 (homozygote, −/−); TM2, TALEN-*Ttc36* male mouse#2 (heterozygote, +/−) TF, TALEN-*Ttc36* female mouse (wild type, +/+).

**Figure 3:**
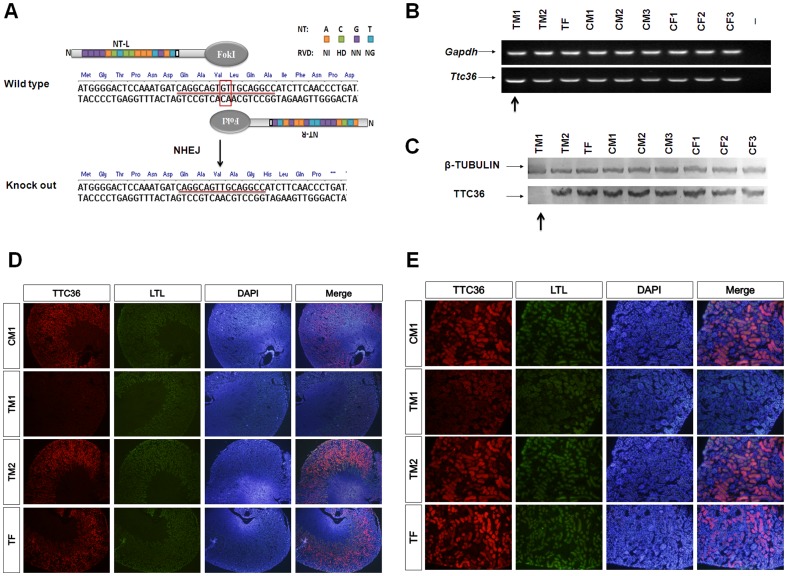
Frame shift induced by TALEN-*Ttc36* and molecular phenotype of mouse kidney. (**A**) Schematic of one mutant frame shift induced by TALEN-*Ttc36* (the first microinjection batch). Translation of TTC36 was stopped with only 15 amino acids remaining in the homozygote because the “GT” base was knocked out. (**B**) Semi-quantitative RT-PCR of *Ttc36* and *Gapdh* (internal control) in knockout mice (TM) with wild type controls (CM and CF). There was a small increase in *Ttc36* mRNA expression in homozygous TM1 (−/−) mice compared to wild type mice (black arrow). (**C**) Western blot for TTC36 and β-tubulin (internal control) in knockout mice (TM) with wild type controls (CM and CF). There was no TTC36 protein expression in homozygous TM1 (−/−) mice (black arrow), and no change in TM2 (+/−). (**D**) and (**E**) Frozen section immunofluorescence showed no TTC36 spatial expression (red) in homozygous TM1(−/−) mice. FITC conjugated LTL (green) for proximal tubules staining and DAPI (blue) for nucleus staining. Image magnification: C, ×100; D, ×400. TM1, TALEN-*Ttc36* male mouse#1 (homozygote, −/−); TM2, TALEN-*Ttc36* male mouse#2 (heterozygote, +/−) TF, TALEN-*Ttc36* female mouse (wild type, +/+). CM1, CM2, CM3, male wild type control mice #1, #2 and #3, respectively; CF1, CF2, CF3, female wild type control mice #1, #2 and #3, respectively.

Although TALEN-*Ttc36* plasmids exhibited the highest activities at the cellular level, in vitro transcribed mRNAs (10 ng/µl) that encoded TALENs were injected into the paternal pronuclei in one-cell embryos to achieve maximum knockout efficiency (Figure 2A). The PCR products (436 bp) from mouse tail were cloned and sequenced to identify mutants. The sequencing results from tails revealed that there were 2 founders (F0) in 3 survived mice: a homozygote-mutant male mouse (TM1, −/−), a heterozygous mutant male mouse (TM2, +/−) and a wild type female mouse (TF, +/+; Figure 2B). The NHEJ repairing induced by TALEN-*Ttc36* resulted in loss of two base pairs, G and T, and the translation of TTC36 was stopped with only 15 remaining amino acids (Figure 3A). In order to investigate the mutagenesis efficiency and compare microinjections in cytoplasm and pronuclei, a second batch of microinjections were performed and 15 new mutant mice were generated, including 2 homozygotes (Figure S1, Table 1). These data demonstrate that cytoplasm microinjection may result in a higher efficiency (85.7%) than that of pronucleus microinjection (30.4%). The second batch provided one additional homozygous mutant besides the TM1 in the first microinjection batch (Table 1). In addition, insertional mutations were only observed in two cases (1–2 and 1–5 in Figure S1) when using cytoplasmic microinjection and both were with the “G” base. Furthermore, mosaicisms also occurred more frequently using cytoplasmic microinjection compared to pronucleus injection.

**Table 1:** TALEN-mediated *Ttc36* gene mutants in C57BL/6J mice.

Microinjection procedure	Transferred embryos (Recipients)	Newborns (birth rate; n (%))	Founders (mutation rate; n (%))	Homozygous mice (biallelic mutation rate; n (%))	Mice with Mosaicism (rate; n (%))
Pronuclei (1^st^)	133 (6)	6 (4.5%)*	2 (33.3%)	1 (16.7%)	0
Pronuclei (2^nd^)	275 (14)	17 (6.2%)	5 (29.4%)	1 (5.9%)	0
Cytoplasm	212 (10)	14 (6.6%)	12 (85.7%)	1 (7.1%)	4 (33.3%)
∑_Pronuclei_	408 (19)	23 (5.6%)	7 (30.4%)	2 (8.7%)	0
∑	620 (49)	37 (6.0%)	19 (51.3%)	3 (8.1%)	4 (10.8%)

Abbreviations: 1^st^, the first microinjection batch; 2^nd^, the second microinjection batch.

Pups were genotyped using sequence analysis of PCR products; this included all pups that lived or died. *Three pups were cannibalized at birth.

### Analysis of the kidney phenotypes using unilateral nephrectomy in vivo

In order to detect the changes and verify the outcomes of the knockout strategy, unilateral nephrectomy was performed on 6-week-old founder mice (F0) and wild-type controls. The left kidneys were removed, and no difference was observed on the surface of kidneys from the TALENs or wild type groups. One left kidney was divided into three parts for genomic DNA, mRNA and protein preparation (Figure 2A). To confirm the gene mutant in the kidney and to avoid mosaicism, the mouse kidney genome PCR products (436 bp) were sequenced. The data showed that the analyzed mouse kidney genome was consistent with that of the mouse tail (Figure 2C). Semi-quantitative RT-PCR revealed a small increase in *Ttc36* mRNA in homozygotes (Figure 3B), and Western blot analysis showed that TTC36 protein expression was absent in the homozygous mouse kidney, but that there was no change in the heterozygous mouse kidney (Figure 3C). Immunophenotyping demonstrated spatially absent TTC36 expression in homozygous the mouse kidney compared with staining of Lotus Tetragonolobus Lectin (LTL), a renal proximal tubule marker (Figure 3D, E). These results also confirmed that TTC36 was expressed in the mouse adult renal proximal tubules, which could represent a new proximal tubule marker. The remaining kidney samples were sent for transcriptomic and proteomics analysis, and these results will be presented in the future.

### Normal reproduction in knockout mice after unilateral nephrectomy and the inherited mutation

**Figure 4:**
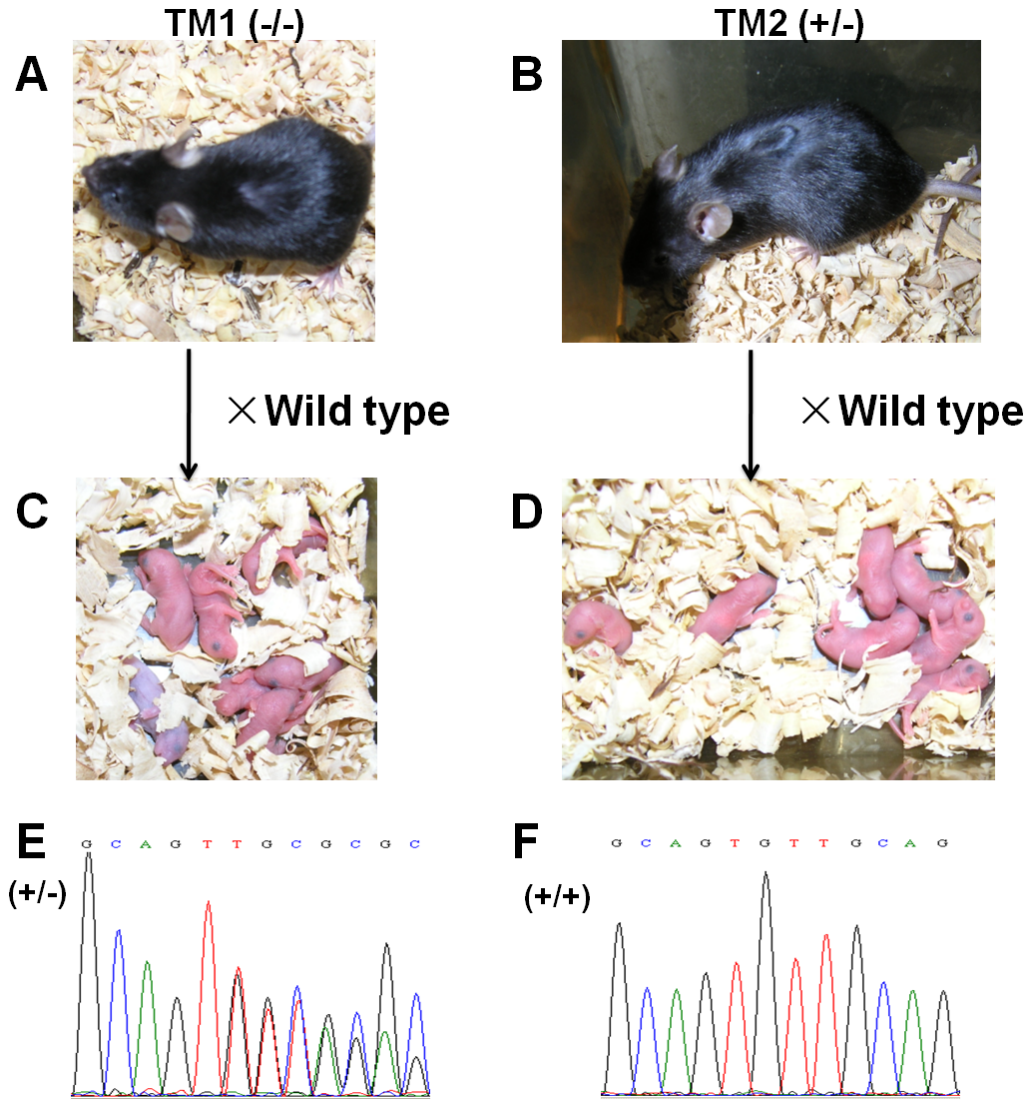
The mutations were inherited stably after unilateral nephrectomy. (**A**) Homozygous (TM1, −/−,) and (**B**) heterozygous (TM2, +/−) mice were mated with C57BL/6 wild-type mice. Founders (F1) and their pups (**C**, **D**) were genotyped using sequence analysis of PCR products. (**E**) and (**F**) DNA sequencing chromatogram of wide type (+/+) and heterozygous pups (+/−) from directed PCR product sequencing.

After unilateral nephrectomy, founders (F0) and wide type controls were mated and the dams gave birth to their offspring. Both TM1 (−/−) and TM2 (+/−) had 7 pups and the genomic analysis, conducted by sequencing PCR products, showed that all the TM1 and TM2 offspring of 7 pups were heterozygous, presenting the same mutation as in *Ttc36* (Table 2, Figure 4). This indicates that unilateral nephrectomy had no effect on the reproduction in knockout mice and that the TALEN-introduced *Ttc36* mutation was stably inherited.

**Table 2:** Overview of TALEN-mediated *Ttc36* gene mutant founder line.

Founders(F0)	All pups	All Founders (F1)	Surviving founders (F1)	Survival rate
TM1 (−/−)	7	7	6	85.7%
TM2 (+/−)	7	2	2	100%
∑	14	9	8	88.9%

Pups were genotyped using sequence analysis of PCR products; this included all pups that lived or died.

## Discussion

The TALEN technology is highly efficient and has been widely used for targeted gene disruption in vivo in recent years. Here, we used a new kidney-specific gene, *Ttc36,* as an example to generate knockout mice for the further investigation of this gene's potential function in the kidney. We created homozygous knockout mice with an inheritable mutation, indicating the technology's high efficiency in targeting DSBs. In addition, using the TALEN technology resulted in a frame shift mutation, which has also been described in other research [5], [10], [11]. However, we completed this research in a total of only 17 weeks, which is less than the expected 18 weeks that was reported by Benedikt Wefers [5]. Time may have been saved during the construction and the TALENS in vitro efficiency testing steps. Moreover, we presented three important additional findings: (1) an improved TALENs efficacy testing system; (2) a comparison between microinjection into cytoplasm and pronuclei; and (3) a novel rapid strategy for generating kidney-associated gene knockouts using unilateral nephrectomy.

An efficiency test should usually be performed before in vitro microinjection at the cellular level. However, the conventional TALENs screening systems were based on β-galactosidase or a single fluorescent reporter system [5], [8]. The efficiency of the former was indirectly determined by the β-galactosidase activity in cell lysates, while the latter was determined through visual observation that did not permit quantitative assessment of transfection efficiency. Here, we have designed a dual-fluorescent reporter system to directly determine the efficiency of candidate TALENs to detect DSBs. Green fluorescence indicated the DSBs efficiency and red fluorescence indicated transfection efficiency. In addition, this simple and practical system could allow data acquisition within 48 h, and could be applied to other similar in vitro Engineered Endonuclease (EEN) efficiency testing, such as ZNF or CRISPR [10], [14]. Furthermore, research using this system furthers our understanding of the TALENs mechanisms and efficiency at the cellular level in vitro and it may play a role in developing new EEN.

TALENs-induced homologous recombination (HR) for targeted gene disruption in vivo was reported by Benedikt Wefers [5], and it involved microinjection of a TALEN mRNA and donor DNA mixture into pronuclei. However, the efficiency between cytoplasmic and pronucleus microinjections was not examined. Our data demonstrated that cytoplasmic microinjection had a higher efficiency (85.7%) than that in pronuclei (30.4%), and the latter had one more homozygous mutant, a TM1 in the first microinjection batch (Table 1). In addition, insertional mutations were only observed in two cases by cytoplasm microinjection (1–2 and 1–5, Figure S1), and both involved the “g” base. The data also demonstrated that pronucleus microinjection generated a more stable natural mutation, which had been previously reported as mosaicism [15], [16]. These results suggest that changes in TALEN microinjection might decrease or avoid the mutant mosaics.

Previous studies have indicated that loss of one kidney, such as in unilateral nephrectomy, can be normally compensated by the other [17]. An adult mouse kidney is about 0.3 gram and use of this organ is sufficient for genomics, transcriptomics and proteomics studies without sacrificing the knockout mice [18], [19]. Thus, unilateral nephrectomy was used to collect the left kidney and to quickly determine the suitability of F0 mice in kidney gene research; preliminary kidney data was subsequently obtained. The kidneys from *Ttc36* homozygous and heterozygous knockout mice were sufficient for use in genotyping, RT-PCR, Western blot and immunohistochemistry to analyze the real changes in the F0 mouse kidney, and this technique has an advantage over traditional embryonic stem cell engineering. This approach is feasible and cost-saving, and it demonstrates that, after recovering from the surgery, mice still have reproductive capacity, which may facilitate future *Ttc36* gene function studies in the kidney.

## Supporting Information

Figure S1 ***Ttc36*** **mutant sequence information in the second microinjection batch.** (**A**) Sequences obtained from mutant mice generated by microinjection of TALEN-*Ttc36* mRNAs into the cytoplasm. (**B**) Sequences obtained from mutant mice generated by microinjection of TALEN-*Ttc36* mRNAs into the pronuclei. The TALEN-binding DNA sequences are underlined. Nucleotide mutations and insertions are shown in lower case and highlighted in red. The wild type TTC36 protein reading frame is shown above and the mutant amino acid sequences are shown on the right of the DNA sequences. Stop codons are shown as “-”.
(TIF)

Sequence S1 **Amino acids sequence of TALEN-Ttc36 pair RVD.**
(DOC)
