# Supplementary figures and images for: A Modified TALEN-Based Strategy for Rapidly and Efficiently Generating Knockout Mice for Kidney Development Studies
Source: PLoS One. 2014 Jan 8;9(1):e84893. doi: 10.1371/journal.pone.0084893 (PMC3885652; doi:10.1371/journal.pone.0084893)

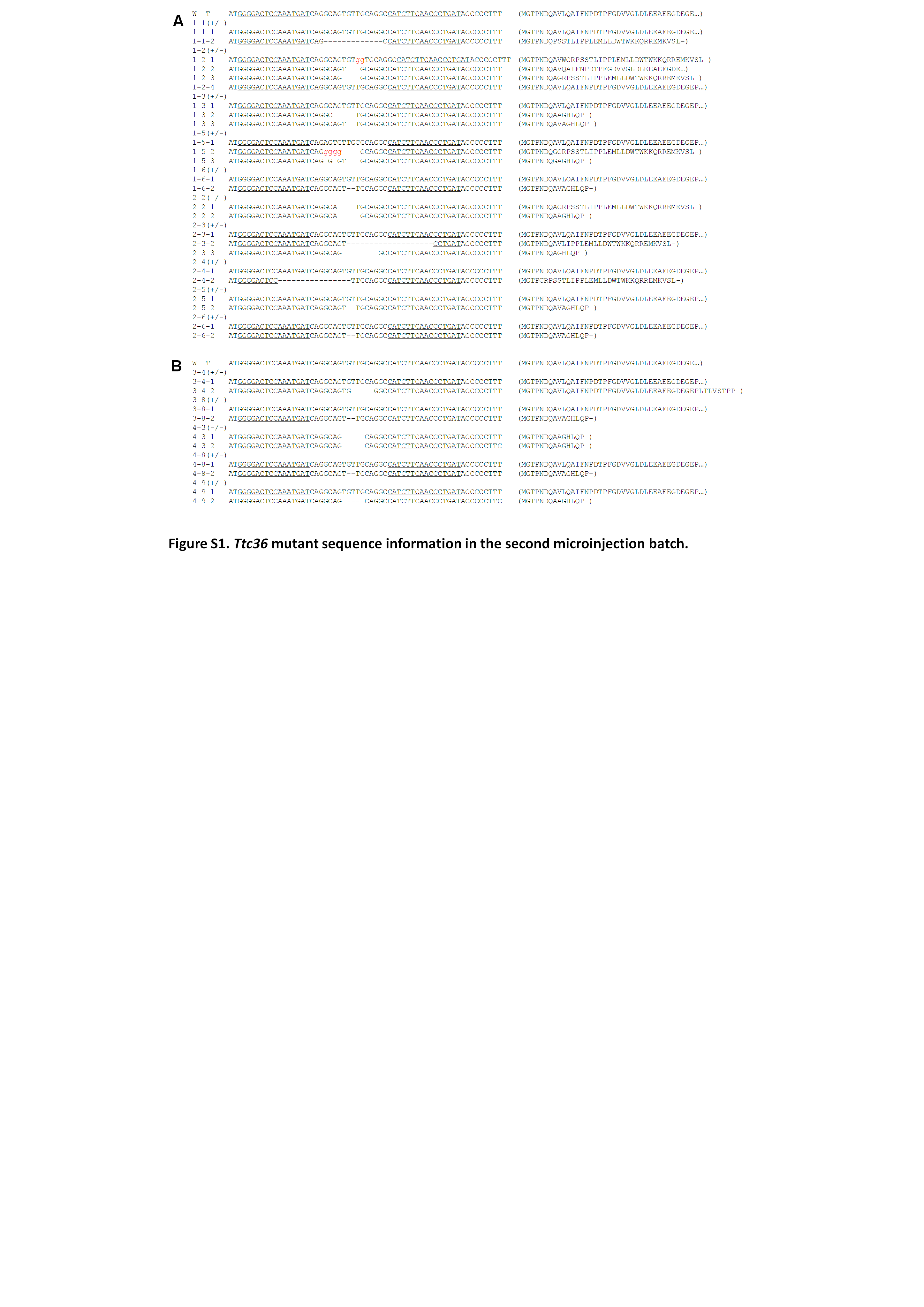

Supplement: Figure S1 — Ttc36 mutant sequence information in the second microinjection batch. (A) Sequences obtained from mutant mice generated by microinjection of TALEN-Ttc36 mRNAs into the cytoplasm. (B) Sequences obtained from mutant mice generated by microinjection of TALEN-Ttc36 mRNAs into the pronuclei. The TALEN-binding DNA sequences are underlined. Nucleotide mutations and insertions are shown in lower case and highlighted in red. The wild type TTC36 protein reading frame is shown above and the mutant amino acid sequences are shown on the right of the DNA sequences. Stop codons are shown as “-”. (TIF) [file pone.0084893.s001.tif]
