# Supplementary material for: A Modified TALEN-Based Strategy for Rapidly and Efficiently Generating Knockout Mice for Kidney Development Studies
Source: PLoS One. 2014 Jan 8;9(1):e84893. doi: 10.1371/journal.pone.0084893 (PMC3885652; doi:10.1371/journal.pone.0084893)
Supplement: Sequence S1 — Amino acids sequence of TALEN-Ttc36 pair RVD. (DOC) [file pone.0084893.s002.doc]

**Sequence S1. Amino acids sequence of TALEN*-Ttc36* pair RVD**

The complete sequence of the vector encoded TALEN-*Ttc36* repeat variable diresidue (RVD) arrays is shown below (RVDs are underlined):

TALEN*-Ttc36 F*

…MEAVHASRNALTGAPLNLTPDQVVAIASNNGGKQALETVQRLLPVLCQDHGLTPDQVVAIASNNGGKQALETVQRLLPVLCQDHGLTPDQVVAIASNNGGKQALETVQRLLPVLCQDHGLTPDQVVAIASNNGGKQALETVQRLLPVLCQDHGLTPDQVVAIASNIGGKQALETVQRLLPVLCQDHGLTPDQVVAIASHDGGKQALETVQRLLPVLCQDHGLTPDQVVAIASNGGGKQALETVQRLLPVLCQDHGLTPDQVVAIASHDGGKQALETVQRLLPVLCQDHGLTPDQVVAIASHDGGKQALETVQRLLPVLCQDHGLTPDQVVAIASNIGGKQALETVQRLLPVLCQDHGLTPDQVVAIASNIGGKQALETVQRLLPVLCQDHGLTPDQVVAIASNIGGKQALETVQRLLPVLCQDHGLTPDQVVAIASNGGGKQALETVQRLLPVLCQDHGLTPDQVVAIASNNGGKQALETVQRLLPVLCQDHGLTPDQVVAIASNIGGKQALETVQRLLPVLCQDHGLTPDQVVAIASNGGGKQALESIVAQL…

TALEN*-Ttc36 R*

…MEAVHASRNALTGAPLNLTPDQVVAIASNIGGKQALETVQRLLPVLCQDHGLTPDQVVAIASNGGGKQALETVQRLLPVLCQDHGLTPDQVVAIASHDGGKQALETVQRLLPVLCQDHGLTPDQVVAIASNIGGKQALETVQRLLPVLCQDHGLTPDQVVAIASNNGGKQALETVQRLLPVLCQDHGLTPDQVVAIASNNGGKQALETVQRLLPVLCQDHGLTPDQVVAIASNNGGKQALETVQRLLPVLCQDHGLTPDQVVAIASNGGGKQALETVQRLLPVLCQDHGLTPDQVVAIASNGGGKQALETVQRLLPVLCQDHGLTPDQVVAIASNNGGKQALETVQRLLPVLCQDHGLTPDQVVAIASNIGGKQALETVQRLLPVLCQDHGLTPDQVVAIASNIGGKQALETVQRLLPVLCQDHGLTPDQVVAIASNNGGKQALETVQRLLPVLCQDHGLTPDQVVAIASNIGGKQALETVQRLLPVLCQDHGLTPDQVVAIASNGGGKQALETVQRLLPVLCQDHGLTPDQVVAIASNNGGKQALESIVAQL…

Note:

TAL effector motif1 (LTPDQVVAIASNIGGKQALETVQRLLPVLCQDHG) to recognize A

TAL effector motif2 (LTPDQVVAIASNGGGKQALETVQRLLPVLCQDHG) to recognize T

TAL effector motif3 (LTPDQVVAIASHDGGKQALETVQRLLPVLCQDHG) to recognize C

TAL effector motif4 (LTPDQVVAIASNNGGKQALETVQRLLPVLCQDHG) to recognize G
